# 1410. Isoniazid Therapy for Latent Tuberculosis Infection in Patients with Cancer Treated with Checkpoint Inhibitors Immunotherapy

**DOI:** 10.1093/ofid/ofab466.1602

**Published:** 2021-12-04

**Authors:** Alexandre Malek, Patrick Chaftari, Pablo C Okhuysen, Hiba Dagher, Harrys A Torres, Ray Y Hachem, Anne-Marie Chaftari, George Viola, Dimitrios P Kontoyiannis, Victor E Mulanovich, Issam I Raad

**Affiliations:** 1 UT MD Anderson Cancer Center, Houston, Texas; 2 UT MDAnderson Cancer Center, Houston, TX; 3 The University of Texas MD Anderson Cancer Center, Houston, Texas, Houston, Texas; 4 The University of Texas MD Anderson Cancer Center, Houston, TX; 5 MD Anderson Cancer Center, Houston, TX; 6 The University of texas MD Anderson Cancer Center, Houston, TX; 7 University of Texas MD Anderson Cancer Center, Houston, TX

## Abstract

**Background:**

Data on the efficacy and tolerability of latent tuberculosis infection (LTBI) treatment in cancer patients receiving checkpoint inhibitor immunotherapy (CPI) is limited. We sought to assess LTBI therapy and its adverse events and outcomes in patients treated with CPI.

**Methods:**

We performed a retrospective cohort study at MD Anderson Cancer Center of adult patients, between April 2016 and May 2021, who were receiving CPI and were diagnosed with LTBI based on positive T-SPOT TB test. We then compared those patients treated with isoniazid (INH) 5mg/kg daily versus those that did not receive INH therapy.

**Results:**

We identified 35 patients treated with CPI who had a diagnosis of LTBI. Patients were divided into 2 groups: CPI alone (23 patients, 65.7%) and CPI+INH (12 patients, 34.3%). The majority of patients in both groups had renal cell carcinoma (34.3%) and melanoma (17.1%). Nivolumab as monotherapy was the most commonly used CPI agent in both groups (37.1%), whereas nivolumab and ipilimumab combination was mainly used in the CPI group (34.7%) compared to CPI+INH group (8.3%). In the CPI+INH group, 7 patients (58.3%) developed moderate to severe hepatoxicity that led to discontinuation of INH and CPI therapy versus none in the CPI group (p= 0.001). There was no statistically significant difference in the alanine aminotransferase (ALT) at baseline between both groups (p=0.117), whereas the median ALT level was significantly higher during CPI+INH therapy compared to CPI alone (135 U/L vs 24 U/L respectively, p=0.025. Furthermore, immune-related adverse events were reported in a total of 12 of 35 patients (34.2%). None of the patients in either group developed tuberculosis reactivation during a follow up period of up to 1148 days.

**Conclusion:**

Our data suggest that latent tuberculosis reactivation is rare in cancer patients on CPI immunotherapy. Hepatotoxicity remains a concern in this patient population with LTBI treated with CPI and INH. With the widespread use of CPI, close laboratory and clinical monitoring is required to avoid life-threatening drug-induced liver injury and interruption of LTBI therapy and immunotherapy. Further clinical studies are warranted to determine the optimal management of LTBI during CPI therapy.

**Disclosures:**

**Pablo C. Okhuysen, MD, FACP, FIDSA**, **Deinove Pharmaceuticals** (Grant/Research Support)**Ferring Pharmaceuticals** (Consultant)**Melinta Therapeutics** (Grant/Research Support)**Merck & Co.** (Grant/Research Support)**Napo Pharmaceuticals** (Consultant, Scientific Research Study Investigator, Research Grant or Support)**Singulex** (Consultant)**Summit Therapeutics** (Grant/Research Support) **Dimitrios P. Kontoyiannis, MD**, **Astellas** (Consultant)**Cidara Therapeutics** (Advisor or Review Panel member)**Gilead Sciences** (Consultant, Grant/Research Support, Other Financial or Material Support, Honoraria)

